# Immunogenic Cell Death in Electroporation-Based Therapies Depends on Pulse Waveform Characteristics

**DOI:** 10.3390/vaccines11061036

**Published:** 2023-05-29

**Authors:** Tamara Polajžer, Damijan Miklavčič

**Affiliations:** Faculty of Electrical Engineering, University of Ljubljana, Tržaška 25, 1000 Ljubljana, Slovenia; tamara.polajzer@fe.uni-lj.si

**Keywords:** electroporation, pulse duration, pulse type, immunogenic cell death, DAMP, ATP, HMGB1, calreticulin

## Abstract

Traditionally, electroporation-based therapies such as electrochemotherapy (ECT), gene electrotransfer (GET) and irreversible electroporation (IRE) are performed with different but typical pulse durations—100 microseconds and 1–50 milliseconds. However, recent in vitro studies have shown that ECT, GET and IRE can be achieved with virtually any pulse duration (millisecond, microsecond, nanosecond) and pulse type (monopolar, bipolar-HFIRE), although with different efficiency. In electroporation-based therapies, immune response activation can affect treatment outcome, and the possibility of controlling and predicting immune response could improve the treatment. In this study, we investigated if different pulse durations and pulse types cause different or similar activations of the immune system by assessing DAMP release (ATP, HMGB1, calreticulin). Results show that DAMP release can be different when different pulse durations and pulse types are used. Nanosecond pulses seems to be the most immunogenic, as they can induce the release of all three main DAMP molecules—ATP, HMGB1 and calreticulin. The least immunogenic seem to be millisecond pulses, as only ATP release was detected and even that assumingly occurs due to increased permeability of the cell membrane. Overall, it seems that DAMP release and immune response in electroporation-based therapies can be controlled though pulse duration.

## 1. Introduction

Electroporation is a phenomenon in which the cell plasma membrane is affected. Electroporation can cause transient change in membrane integrity followed by survival of the cells (termed reversible electroporation, RE) or cell death (termed irreversible electroporation). Over the last decades, therapeutical benefits of electroporation were widely recognized. Today, the best-known electroporation-based therapies in medicine are electrochemotherapy (ECT), gene electrotransfer (GET) and irreversible electroporation (IRE). Electrochemotherapy (ECT) is a local cancer treatment that combines chemotherapeutic agents with high-voltage electrical pulses [1,2]. GET or transfection by electroporation is a method of introducing foreign nucleic acids into cells to produce genetically modified cells, used for DNA vaccines delivery, gene therapy and ex vivo engineering [3,4,5]. IRE is a method of tissue ablation, used for cancer [6] and heart arrythmia [7,8] treatment.

In theory, electroporation is categorized as RE or IRE, however, in practice, RE and IRE overlap, i.e., are both present [9,10]. There is always IRE present in addition to RE (including in ECT and GET), and there is always electroporation in addition to drug/DNA delivery. In in vivo applications of ECT and GET, cells are exposed to different electric field intensities due to inhomogeneous electric field distribution. The highest electric field is in the proximity of the electrodes, leading to cell death caused only by electric pulses, i.e., IRE. Furthermore, electric pulses are the basis for drug/DNA delivery, ECT and GET, and, therefore, cells exposed to pulses will respond to pulses, regardless of the presence or absence of therapeutical agents. Understanding the cell response to electric pulses from the perspective of IRE is therefore also important for ECT and GET. The efficacy of electroporation depends on parameters such as pulse duration (milliseconds, microseconds, nanoseconds), number of pulses, frequency, strength of the electric field and pulse type (monopolar or bipolar). Traditionally, every electroporation-based therapy is performed with its typical pulse duration. GET is performed with milliseconds-long pulses [3]. In ECT, somewhat shorter—100 microseconds long—pulses are used [1]. Such pulse duration is also used in IRE, but a higher number of pulses and/or a higher electric field is applied [6] to reach the range of IRE. Even though treatment with long monopolar pulses used in GET, ECT and IRE is very successful, it has some disadvantages. Such treatment causes pain and muscle contractions. To overcome the disadvantage of IRE, a new type of pulses called high frequency irreversible electroporation (HFIRE) has been introduced. In HFIRE electroporation, 100 μs long pulses were replaced by a series of 1–10 μs long pulses of alternating polarity [11]. Today, HFIRE pulses are the prevalent pulse type used in cardiac tissue ablation used for elimination or isolation of the sources of arrhythmic triggers, thereby treating abnormal heart rhythm, e.g. atrial fibrillation [8,12].

A special group of pulses are present as nanosecond pulses (nsPEF). They have several potential advantages in electroporation-based procedures over the conventional micro- and millisecond pulses, such as reduced electrochemical reactions and reduced muscle contractions, making them alluring for use in biomedicine and the food industry. Interestingly, nsPEF can affect the membranes of intracellular organelles and trigger intracellular effects, whereas longer micro- and millisecond pulses have more profound effect on the cell plasma membrane [13,14]. Affecting the intracellular membrane has made nsPEF the most investigated pulse parameter with respect to cell death caused by electroporation [15].

Electroporation studies in the last two years have shown that ECT, GET and IRE can be successfully achieved with virtually any pulse duration (millisecond, microsecond, nanosecond) and pulse type (monopolar, bipolar). Based on available preclinical data, ECT can be equally effective using nsPEF [16,17,18] and HFIRE pulses [19]. GET can be performed with nsPEF [20] and HFIRE pulses [21], however, such pulses are significantly less efficient in achieving GET compared to longer monopolar pulses (e.g., 100 μs and ms). Furthermore, it was confirmed that IRE can be achieved with any pulse duration in the range of a few ns to ms and any pulse type [22,23]. Nevertheless, whether the mechanisms of electroporation and treatment response to different pulse durations and types are the same or different remains unknown.

Electroporation-based therapies in addition to the main goal of the therapy (transient change in cell permeability or cell death) also activate the immune system. While the electrical pulses are the only therapeutic means in IRE, in ECT and GET, therapeutical agents (chemotherapeutics and genetic material) are also added in the treatment. Therefore, cell response in ECT and GET can be due to electrical pulses or therapeutical agents alone or due to their synergistic affect. Electroporation treatment can lead to inflammation and activation of the immune system, which is not always desirable. Immune response is favorable when electroporation is used for cancer treatment in reversible and irreversible electroporation [24,25]. On the contrary, immune response is not desirable in ablation of cardiac tissue because inflammation prolongs healing of the treated cardiac tissue [26,27]. In GET, immune response is only desired for DNA vaccine therapies. In gene therapies, activation of the immune system can destroy transfected cells and prevent the formation of transgenic proteins, so high immune response is highly undesirable [28]. Understanding how electroporation treatment affects activation of immune response is of importance, even though only cell response to electric pulses is taken into consideration, as this understanding can be used to improve the outcome of the electroporation-based therapy.

Activation of immune response can be caused by immunogenic cell death in which damage-associated molecular patterns (DAMP molecules) are released from the cell. In the extracellular space, DAMP molecules are recognized by cells of the immune system, which triggers a cascade of events leading to the removal of damaged or dying cells, activation of specific memory cells for long-lasting tumor protection and destruction of metastases [29]. So far, activation of immune response through the release of DAMP molecules (such as ATP-adenosine triphosphate, HMGB1-High mobility group box 1, calreticulin, nucleic acids, uric acid) has been confirmed in electroporation studies of ECT [24], GET [30] and IRE [31,32]. Furthermore, DAMP molecules were detected also when using HFIRE pulses [33] and nsPEF [34,35,36]. The release of DAMP molecules increases with increasing pulse amplitude, number and duration. Furthermore, our previous study has shown a strong correlation between the release of DAMP molecules and survival (irreversible electroporation), while the correlation between the release of DAMP molecules and reversible electroporation was weak or even non-existent [31]. Therefore, a very low presence of passively released DAMP molecules or the absence of actively released DAMP molecules is expected after application of reversible electroporation. However, in vivo electric pulses in reversible electroporation (ECT and GET) in practice often cause irreversible electroporation as well. This occurs in the close proximity of the electrodes due to inhomogeneous electric field distribution [9]. Therefore, even an application of reversible electroporation (ECT and GET) will result in some immune response, regardless of the presence of the therapeutic agent. While recent studies have shown that any electroporation-based therapy can be achieved with any pulse duration or pulse type, it remains unknown if activation of immune response depends on pulse duration and pulse type. Therefore, the release of DAMP molecules (ATP, HMGB1 and calreticulin) in response to different pulse durations and pulse types was investigated in this study. Three cell lines were used to investigate if DAMP release due to electroporation is cell type dependent. Since we have previously shown that DAMP molecules are strongly correlated with cell death, experimental points for each pulse duration and type were determined based on survival curves. Electroporation parameters causing 90, 50 and 20% of survival were used in assessing DAMP release.

## 2. Materials and Methods

Cell preparation: All cell lines used in this study are from the European Collection of Authenticated Cell Cultures (Chinese hamster ovary—CHO cat #85051005, mouse melanoma cells—B16F1 cat #92101023 and rat heath myoblast—H9c2 cat #88092904). B16F1 and H9c2 were grown in DMEM growth medium supplemented with 10% fetal bovine serum (Sigma Aldrich, ZDA, St. Louis, MO, USA), L-glutamine (StemCell, Vancouver, BC, Canada) and antibiotics penicillin/streptomycin (PAA) and gentamycin (Sigma Aldrich, ZDA). The CHO cells were grown in HAM-F12 growth medium supplemented with 10% fetal bovine serum, L-glutamine and antibiotics penicillin/streptomycin and gentamycin. Cells were subcultured every 3–4 days and incubated at 37 °C in a humidified atmosphere with a 5% CO_2_ incubator. After reaching 70% confluency, cells were detached with trypsin solution (10 × trypsin-EDTA (PAA, Leonding, Austria) and 1:9 diluted in Hank’s basal salt solution (StemCell, Canada). Trypsin was inactivated after 2–3 min by the growth medium. Cells were centrifuged for 5 min at 180 g and 22 °C, then supernatant was removed. Cells were mixed with the growth medium to a certain cell density. Cells were then transferred to 2 mm cuvettes. For pulse durations of 5 ms, 100 μs and 200 ns and HFIRE pulse type, 150 μL of cell suspension with cell density at 1 × 10^6^ cells/mL was used. Due to impedance matching of the 4 ns pulse generator, the sample volume was limited to 60 μL. Therefore, to have the same number of cells for DAMP analysis as with other pulses, cell density was increased to 2.5 × 10^6^ cells/mL.

Electric pulse generation: In this study, four different pulse generators were used. For delivery of 5 ms long pulses (eight pulses delivered with 1 Hz repetition frequency, various electric field strength), a laboratory prototype pulse generator, described in [37], was used. The 100 μs long pulses (eight pulses delivered with 1 Hz repetition frequency, various electric field strength) and HFIRE pulses were delivered with a prototype high-frequency pulse generator, L-POR V0.1 (mPOR, Slovenia), previously described in [20]. In HFIRE pulse protocol, bipolar pulses of 2 μs duration of positive and negative phase were applied. The pause between positive and negative pulse phase and the pause between bipolar pulses were 2 μs. A total of 100 bursts were applied, and in each burst, 32 pulses were delivered. The burst repetition rate was 1 Hz, and various electric field strengths were used. Delivered pulses were monitored by a high-voltage differential probe, HVD3605A (Teledyne LeCroy, Chestnut Ridge, NY, USA), current probe, CP031 (Teledyne LeCroy, USA), and HDO6000 high-definition oscilloscope (Teledyne LeCroy, USA). Pulses of 200 ns duration (100 pulses delivered with 10 Hz pulse repetition frequency, various electric field strength) were delivered by the CellFX System electroporator (Pulse Biosciences, Hayward, CA, USA). Voltage was measured by a 1 kΩ resistor and the Pearson current monitor model 2877 (Pearson Electronics, Palo Alto, CA, USA). Current was measured by the Pearson current monitor model 2878 (Pearson Electronics, USA). Voltage and current measurements were monitored by the oscilloscope WaveSurfer 3024Z, 200 MHz (Teledyne LeCroy, USA). Pulses of 4 ns duration were delivered by the FPG20-1NM4 electroporator (FID Technology, Germany). Pulse measurement was obtained by a high voltage coupler (CPF 30L50-B500-D40, 50 Ohm, DC-500 MHz, FID GmbH, Burbach, Germany) with a division ration of 57.8 dB (1:780). The high voltage coupler was placed between two high-voltage cables (FC26, FID GmbH, Germany), which are delivering pulses from the generator to the cuvette holder. The high voltage coupler was connected with an SMD cable in the –20 dB attenuator (1:10, 50 Ohm, DC-1 GHz, Telegärtner, Steinenbronn, Germany) to the oscilloscope WaveSurfer 3024Z, 200 MHz (Teledyne LeCroy, USA), where pulses and reflected waves were measured. Different numbers of pulses were applied, while electric field strength was fixed at 12 kV and pulses were delivered with 500 Hz pulse repetition frequency. The size of the sample in the 2 mm cuvettes was limited to 60 μL to assure impedance matching between generator and biological load (cuvette). Measured voltage and current of different pulse durations and pulse types are presented in Supplementary Figure S1.

Temperature measurement: The temperature of the sample was measured during pulse delivery with a fiber optic sensor system (opSens, Québec, QC, OTG-M170, Canada). The fiber optic sensor was carefully placed in the cell suspension. The temperature was measured in HAM-F12 growth media with different CHO cell densities present (0 c/mL–no cells, 1 × 10^6^ c/mL and 2.5 × 10^6^ c/mL) for pulse parameters that cause 20% survival. To evaluate thermal damage, the damage index was calculated using the Arrhenius equation, described in [38]. This equation was previously used for thermal damage evaluation in in vitro experiments [39], therefore, some equation parameters needed for our calculations were taken from that study.

Permeabilization assay: Prior to pulse application, cells were mixed with propidium iodide (PI, Life Technologies, Carlsbad, CA, USA) to a final concentration of 100 μg/mL. After three minutes, the sample was removed from the cuvette and the uptake of PI in cells was analyzed by the flow cytometer (Attune NxT; Life Technologies, Carlsbad, CA, USA) using a 488 nm blue laser and 574/26 nm band-pass filter. The analysis of 10,000 events was performed by the Attune Nxt software. On the dot-plots of forward-scatter and side-scatter, the debris and clusters were excluded from the analysis. Fluorescence intensity histograms were used to determine the percentage of PI permeabilized cells. Gating was set according to sham control (0 V). Measurements for each data point were repeated three times.

Viability assay: After pulse application, samples were diluted in growth media to obtain the same concentration (5 × 10^5^ cell/mL) for all the samples. A total of 80 μL of sample was then transferred to a 96-well plate (TPP, Trasadingen, Switzerland) and incubated at 37 °C and in a humidified 5% CO_2_ atmosphere for 24 h. According to manufacturer’s instructions (CellTiter 96 AQueous One Solution Cell Proliferation Assay, Promega, Madison, WI, USA), 20 μL of MTS tetrazolium compound was added to the samples, and after 2 h, the absorbance of formazan (reduced MTS tetrazolium compound) was measured with a spectrofluorometer (Tecan Infinite M200, Tecan, Grödig, Austria) at 490 nm. The percentage of viable cells was obtained by the normalization of sample absorbance to the absorbance of the control (0 V). Each sample was performed in three technical repetitions, and measurements for each sample were repeated three times.

DAMP assays: Based on viability and permeabilization curves for each pulse duration (4 ns, 200 ns, 100 μs, 5 ms) and pulse type (HFIRE), electric field strengths needed for achieving 90, 50 and 20% survival (based on MTS after 24 h) were used for analysis of DAMP molecules release from cells.

ATP assay: After pulse application, samples were diluted in growth media to obtain the same concentration (5 × 10^5^ cell/mL) for all samples. Afterwards, 50 μL of sample was transferred to 100 μL of growth media in a white 96-well plate. For 30 min analysis, 30 min after pulse application, 50 μL of ATP reagent (RealTime GloTM Extracelular ATP Assay, Promega, USA) was added to the samples, the samples were incubated for 1 min at room temperature and then the luminescence signal was measured with the Tecan Infinite M200 spectrophotometer (Tecan, Switzerland). For ATP kinetics analysis, an ATP reagent was added immediately after sample transfer and the luminescence signal was measured for 24 h, every 5 min while the temperature was maintained at 37 °C. Measurements for each data point were repeated at least three times.

HMGB1 assay: After pulse application, samples were diluted in growth media to obtain the same concentration (5 × 10^5^ cell/mL) for all samples. A total of 80 μL of sample was then transferred to a white 96-well plate (TPP, Switzerland) and incubated at 37 °C with a humidified 5% CO_2_ for 4 or 24 h. After incubation, HMBG1 was analyzed with Lumit™ HMGB1 Human/Mouse Immunoassay (Promega, USA) according to manufacturer’s instructions. First, 20 μL of antibody mixture was added to the samples and incubated for 90 min in the dark at room temperature. Second, 25 μL of detection reagent was added to the samples, the samples were incubated for 3–5 min and then luminescence was measured with the Tecan Infinite M200 spectrophotometer. Measurements for each data point were repeated at least three times.

Calreticulin assay: After pulse application, samples were diluted in growth media to obtain the same concentration (5 × 10^5^ cell/mL) for all samples. Afterwards, samples were transferred to a 48-well plate (TPP, Switzerland) and incubated at 37 °C with a humidified 5% CO_2_ for 4 or 24 h. Afterwards, cells were harvested and washed twice (400 g, 5 min) with ice cold PBS buffer (Merck, ZDA; Sigma Aldrich, Germany) with 10% FBS. A total of 50 μL of primary antibody Calreticulin Monoclonal Antibody (Invitrogen, ZDA, Waltham, MA, USA) diluted 1:100 in PBS buffer with 3% FBS was added to samples and incubated for 30 min at 4 °C in the dark. Cells were then washed twice with ice cold PBS buffer with 10% FBS. A total of 50 μL of secondary antibody Goat anti-Mouse IgG, Alexa Fluor 405 (Invitrogen, ZDA) diluted 1:250 in PBS buffer with 3% FBS was added to the cells and incubated for an additional 20–30 min at 4 °C in the dark. Cells were then washed twice with ice cold PBS buffer with 3% FBS and diluted in 50 μL of PBS buffer with 3% FBS. Before analysis, 5 μL of PI was added to the samples, and they were incubated in the dark at room temperature for 15 min. A flow cytometer was used for analysis. A 405 nm violet laser was used for detection of calreticulin with a 440/50 nm band-pass filter, and a 488 nm blue laser with a 574/26 nm band-pass filter was used for detection of PI. By gating process, the presence of calreticulin was analyzed only in viable cells (PI-negative cells). Median fluorescence of calreticulin on viable cells was obtained from a fluorescence intensity histogram, determined as the median value of the measured signal (Figure S2). Measurements for each data point were repeated at least three times.

Statistical analysis: For statistical analysis, we used SigmaPlot 11.0 (Systat Software, San Jose, CA, USA). One-way ANOVA, followed by the Holm–Sidak post hoc test, was performed to determine the statistical significance of differences between control and pulse duration or pulse type.

## 3. Results

### 3.1. Pulse Parameters

Permeabilization and survival curves were obtained using different pulse durations and pulse types (Supplementary Figure S3). This allowed us to determine electric field strengths (in the case of 4 ns pulses, this was a number of pulses) for experimental points leading to 90, 50 and 20% of survival for each pulse duration and pulse type (Supplementary Figure S1). These experimental points (Table 1) were then used for analysis of released DAMP molecules, namely ATP, calreticulin and HMGB1.

### 3.2. Temperature Measurement

Different cell components have different thresholds for thermal damage. Temperatures of 85–90 °C are needed for DNA damage, 40–60 °C for protein damage and 10–40 °C for lipid damage. Thermal damage is determined by thermosensitivity of cells and exposure time. Most commonly, the threshold for thermal damage is considered to be at 43 °C if exposure time is long enough [38]. Temperature measurements during pulse application (Figure S4) for pulses leading to 20% survival, i.e., the highest amplitude/electroporation intensity tested, showed different temperature responses. The 100 μs, HFIRE and 200 ns pulses did not exceed the threshold for thermal damage. The 5 ms pulses exceeded the threshold with the last pulse, overall, for a second, which is probably too short of a period for thermal damage injures. For 4 ns pulses, temperature increased above 50 °C for approximately 8 s, therefore, some results obtained with 4 ns pulses might be related to temperature changes. Overall, however, 8 s is not long and may not be sufficient for severe thermal damage. This was further explored with calculations of thermal damage, where values above 0.53 are considered to cause thermal damage [38,39]. Our calculations showed that pulses used in this study, at the highest dose, i.e., achieving 20% survival, did not cause thermal damage (Ω_5 ms_ = 0.0013609, Ω_100 μs_ = 0.06733, Ω_HFIRE_ = 0.0064332 and Ω_200 ns_ = 0.27324) and (Ω_4_ ns = 0.024311).

### 3.3. DAMP Molecules Analysis

DAMP molecules have been so far investigated at different timepoints after electroporation. To investigate when ATP should be detected in electroporation studies, a kinetics assay (for ATP release into the extracellular space) was performed. After pulse application, ATP release was measured every 5 min for 24 h. Obtained kinetics revealed that ATP is being released into the extracellular space immediately after pulse delivery, and ATP release steadily declined afterwards. Furthermore, ATP is being quickly degraded, as the signal is completely reduced in less than one hour after electroporation, irrespective of electric field intensity (% of survival). The same trend of ATP release was observed for all pulse intensities, i.e., at 90, 50 and 20% survival, but the total values for ATP were the lowest at 90% survival and the highest at 20% survival. This was observed with all pulse durations, all pulse types and in all cell lines (results are shown for B16F1 using pulse amplitudes causing 20% of survival, other results are not presented as the trend of ATP release was similar) (Figure S5).

**Figure 1:**
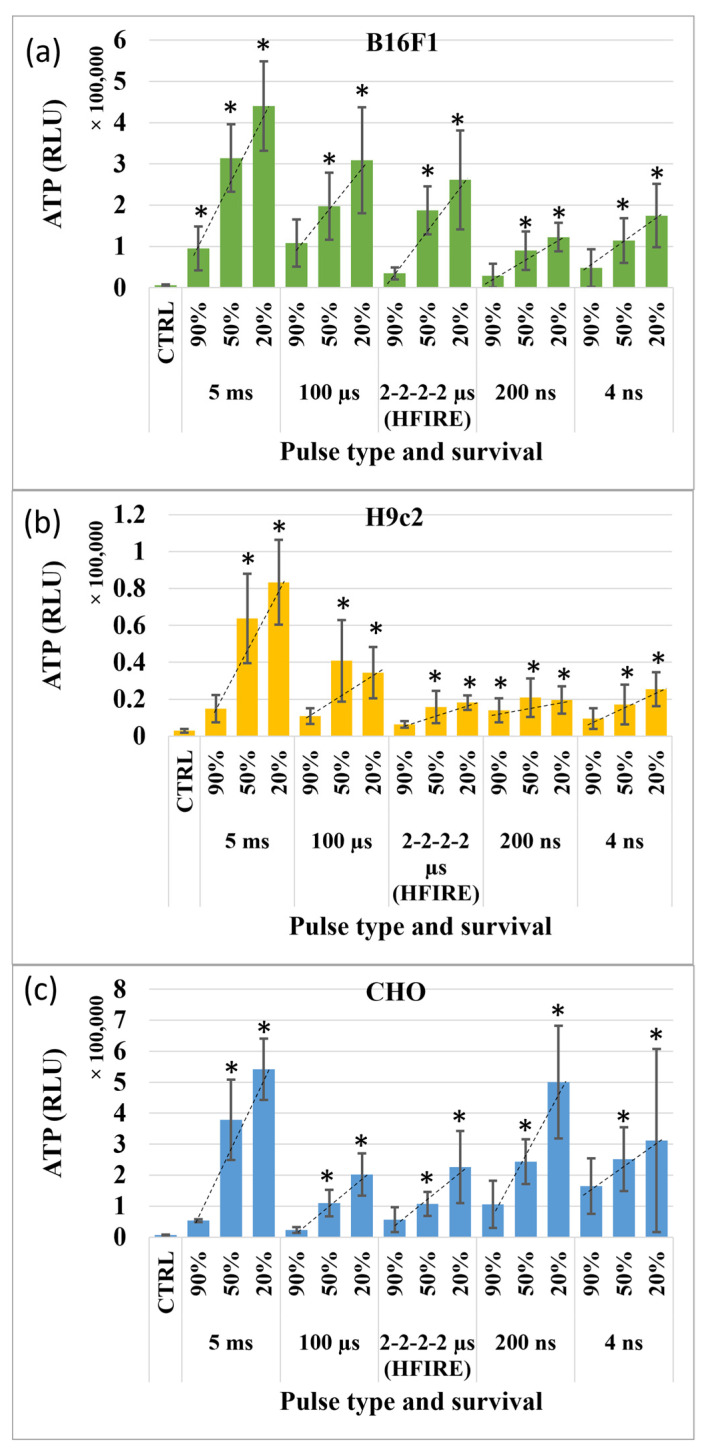
ATP release 30 min after pulse application. Dotted line (…) represents trendline to show the direction of ATP release increase. * marks electroporated samples in which ATP release is statistically significantly different compared to the control. (**a**) B16F1; (**b**) H9c2; (**c**) CHO.

Differences in overall ATP release were detected when different pulse durations and pulse types were used. The highest ATP release was observed in cells exposed to 5 ms, followed by 100 μs, HFIRE, 200 and 4 ns pulses. Therefore, this was more thoroughly investigated at 30 min after pulse application (Figure 1). For every pulse duration and pulse type increase in electric field strength (decrease in survival), it resulted in an increase of ATP release, which indicated that the release of ATP increases with the degree of cell injury. This was observed in all cell lines investigated.

At closer inspection, comparison of released ATP after pulse application using different pulse durations and pulse types shows that ATP release is not always the same, even though the same percentage of cells die. The most ATP is released when millisecond pulses are used, and this goes for all tested cell lines. Other pulse durations and pulse types impact the ATP release differently in different cell lines. For example, in CHO, 200 ns and 4 ns pulse durations induce higher ATP release than microsecond pulses and HFIRE. These results are reversed in B16F1, where microsecond pulses and HFIRE induce higher pulses and nanosecond pulses the lowest. For H9c2, nanosecond pulses as well as HFIRE led to low ATP release. It seems that ATP release is pulse duration, pulse type and cell type dependent.

**Figure 2:**
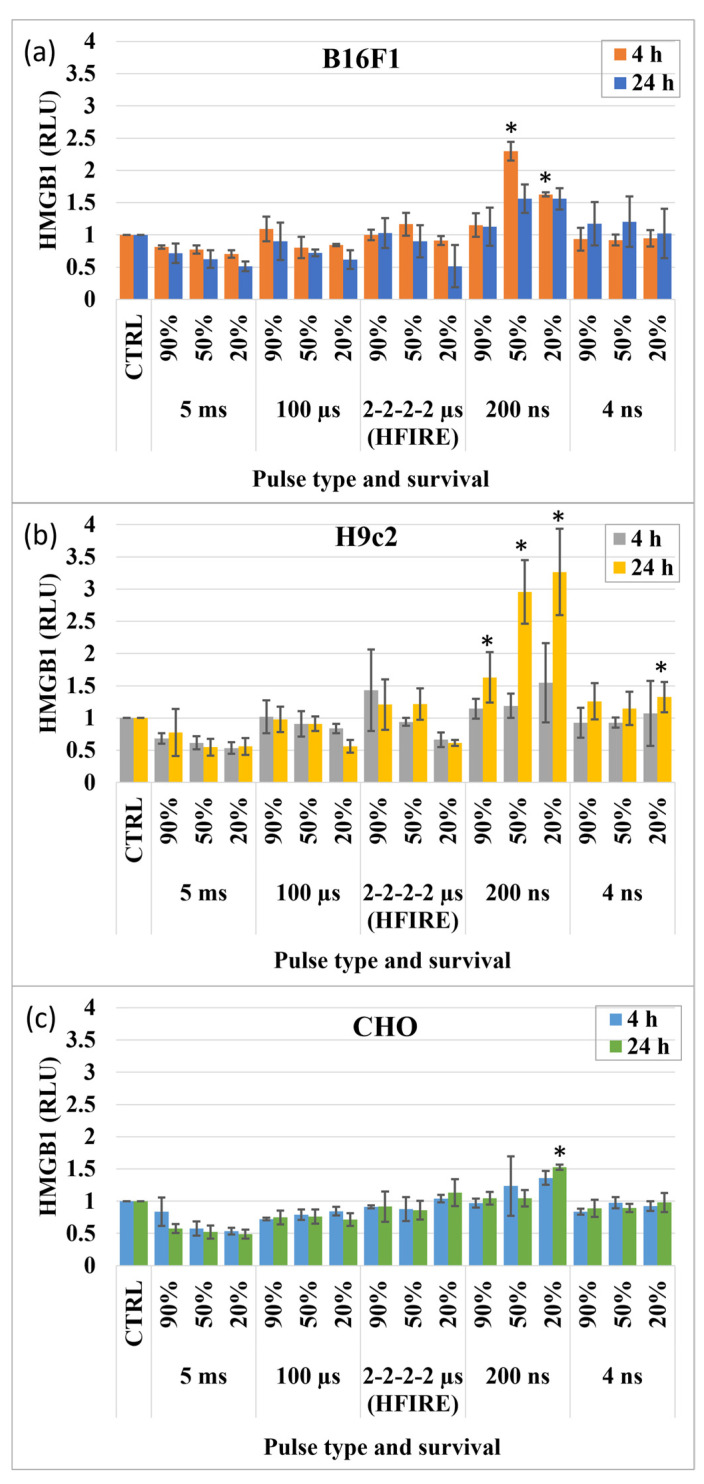
HMGB1 release 4 and 24 h after pulse application. * marks electroporated samples in which HMGB1 release is statistically significantly different compared to the control. (**a**) B16F1; (**b**) H9c2; (**c**) CHO.

The next molecule of interest was nuclear protein HMGB1. Since no kinetics assays are available, the release of HMGB1 into the extracellular space was evaluated at 4 and 24 h after electroporation (Figure 2). In three different cell lines, a statistically significant increase in HMGB1 signal in comparison to control was only observed when pulses with 200 ns duration were used. Furthermore, some differences in the HMGB1 obtained with 200 ns were detected between the cell lines. In the H9c2 cell line, a statistically significant signal increase was detected at 90, 50 and 20% survival; in B16F1, these differences were present only at 50 and 20% survival, while in the CHO cell line, only at 20% survival. HMGB1 was present in all cell lines where survival was the lowest, thus injury was the highest. In addition, in H9c2 cells, HMGB1 was detected also after electroporation with 4 ns pulses, again only at the lowest survival. No statistically significant signal increase in HMBG1 release was detected after millisecond, microsecond or HFIRE pulse treatment in any cell line.

**Figure 3:**
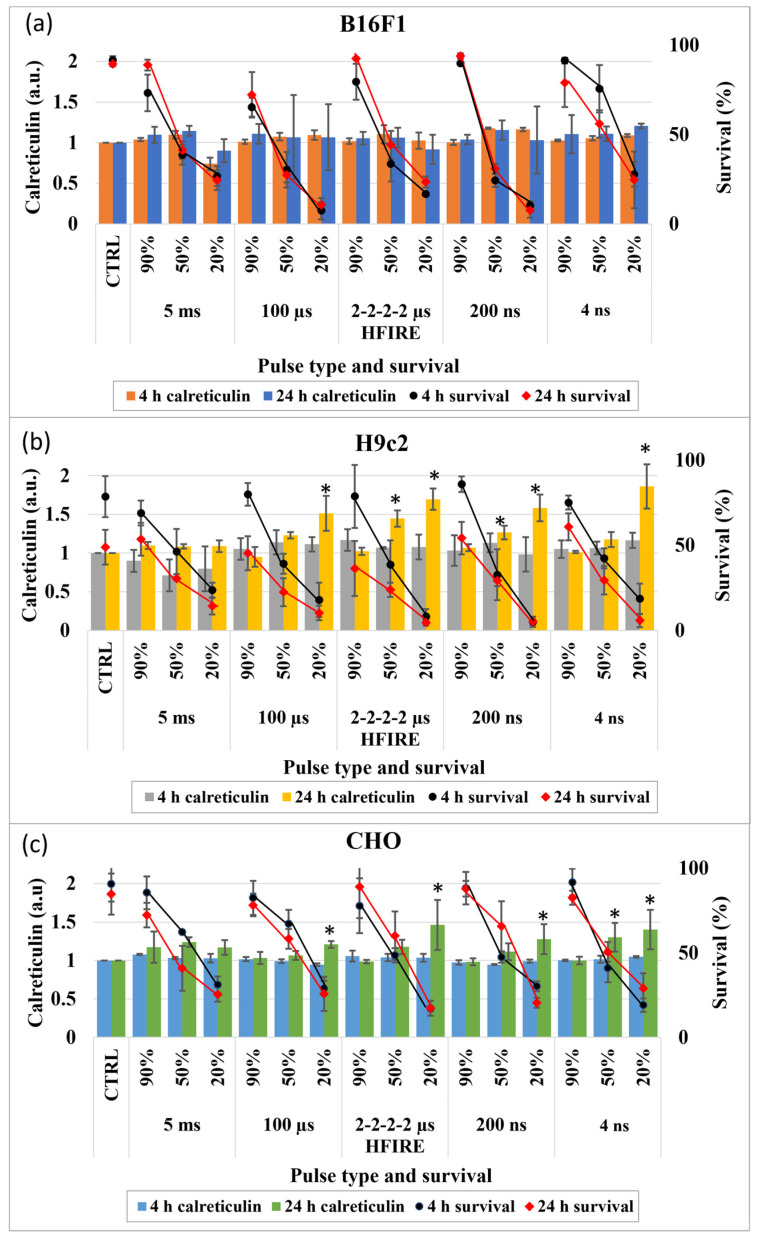
Calreticulin externalization 4 and 24 h after pulse application. Survival curves of live cells detected with PI are also presented (black line for 4 h after pulse treatment and red for 24 h after pulse treatment). * marks electroporated samples in which calreticulin is statistically significantly different compared to the control. (**a**) B16F1; (**b**) H9c2; (**c**) CHO.

The third molecule of interest was calreticulin, which was similar to HMGB1 investigated at 4 and 24 h after electroporation (Figure 3). While externalization of calreticulin was not detected in B16F1 at either 4 or 24 h after pulse application, in CHO and H9c2 cells, the calreticulin was detected 24 h after electroporation. In CHO and H9c2 cells, a statistically significant increase of calreticulin was present after microsecond, nanosecond (200 and 4) and HFIRE pulse application when pulses leading to the lowest survival (20%) were used. Differences between CHO and H9c2 cell lines and pulse durations/types were observed when pulses leading to 50% survival were applied. While the statistically significant increase of calreticulin in CHO was observed when 4 ns pulses were used, in H9c2, this was observed with 200 ns and HFIRE pulses. All signals obtained at 50% survival are weaker than those obtained at 20% survival, therefore, it seems the signal intensity is increasing with the decrease of survival, suggesting that release of DAMPs increases with the degree of injury.

## 4. Discussion

In addition to the transient membrane permeabilization (ECT and GET) and cell death (IRE), electroporation treatment can also trigger immune response. Activation of an inflammatory response and activation of the immune system can be either desired or undesired. It is desired in cancer therapies where ECT, GET and IRE are used, as untreated metastases are destroyed and long-lasting protection against the tumor is established. While in IRE for tumor treatment immune response is desired, it is not desirable in ablation of cardiac tissue because inflammation prolongs healing of the treated cardiac tissue [26,27]. In GET, the presence of immune response such as in IRE can be wanted or unwanted depending on the aim of the treatment. In DNA vaccination therapies, the presence of an inflammatory response and immune response is desired, as it leads to enhanced production of antibodies, thus enhancing the efficiency of vaccination [40,41,42]. However, in gene therapy, activation of immune response is unwanted, as it may destroy the transfected cells and prevent transgenic protein expression [43,44].

Recent studies have shown that ECT, GET and IRE can be achieved not only with traditional pulse parameters, but also with nanosecond or HFIRE pulses. Any pulse duration or pulse type can therefore be used for electroporation-based therapy. In addition, in all therapies, immune response can affect treatment outcome, and the possibility of controlling and predicting an immune response could improve the treatment. So far, activation of the immune system was demonstrated for different electroporation-based therapies, however, activation of the immune system with pulses of different durations including milliseconds (used in GET), microseconds (used in ECT and IRE), nanoseconds and HFIRE pulses (used for IRE) has not yet been systematically addressed. In this study, we investigated if different pulse durations and pulse types cause different or similar activation of the immune system by assessing DAMP release from cells.

Activation of the immune system can be triggered by a special type of cell death, called immunogenic cell death (ICD). In ICD, the key mediators are DAMP molecules [45]. For ICD prediction, DAMP molecules such as ATP, HMBG1 and calreticulin are usually detected, as they are known as the gold standard for predicting ICD in cancer cells [29]. Presence of DAMPs after electroporation has been identified in numerous studies [24,30,31,32,33,34,35,46,47]. In our previous study, we showed that release of DAMP molecules correlates strongly with IRE, although a weaker correlation was also observed with reversible electroporation [31]. Therefore, the experimental points of this study were determined on the survival curve. The first experimental point was 90% of survival, where most of the cells survived but were permeabilized, next was 50% of survival, where half of the population died and the last one was 20% of survival, where most cells died. At these experimental points, the release of gold standard DAMP molecules was investigated at different times after electroporation.

Interestingly, ATP was used for permeabilization detection in the earliest electroporation studies. In our present study, we observed that all ATP is released to the extracellular space immediately after pulse application and no additional ATP is being released in the extracellular space within the next 24 h (Figure S5). In addition, in the extracellular space, ATP is very unstable and the levels of ATP decrease to normal (control) in less than an hour. Such ATP release kinetics seem to be present in all electroporation treatments, i.e., independent on pulse parameters and cell type.

Upon a closer look at ATP release at 30 min after pulse application, we see different ATP release in response to different pulse durations and pulse types (Figure 1). The only consistent difference in ATP release in all cell lines was obtained with millisecond pulses, where ATP release was the highest. However, the three cell lines had different ATP release when microsecond, nanosecond and HFIRE pulses were used, suggesting different pulse durations and pulse types may have triggered different damage responses in different cell lines. Whether the differences, when using different pulse durations and pulse types, are related to different permeabilization mechanisms due to pulse duration or type remains unknown. Furthermore, some of these differences may even be cell type dependent. Nevertheless, all pulse durations, pulse types and cell types have common features. ATP release is increasing with a decrease of survival, probably related to the increase of cell injury (plasma membrane) as the electric field is increasing. This is in line with our previous observations on DAMP molecule release and the correlation between DAMP molecule release and cell death [31].

High-mobility group protein 1 (HMGB1) is an evolutionary conserved nonhistone protein. The cellular location of HMGB1 varies depending on cell type, tissue and stress signals, and its location is key to its functions. HMGB1 is normally located in the nucleus [48]. In the nucleus, HMGB1 acts as a DNA chaperone, where it maintains the structure and function of chromosomes. Under various stress conditions, HMGB1 can shuttle from the nucleus to the cytoplasm and then into the extracellular space. In response to damage, HMGB1 can be actively secreted or passively released into the extracellular space, where it acts as a DAMP molecule—triggering inflammation and activating immune response [49]. The transport of HMGB1 into the extracellular space is triggered by different changes in the cell line posttranslational modifications or activation of cell death pathways [48]. So far, this has been confirmed in apoptosis, pyroptosis, necroptosis and ferroptostis [48]. Interestingly, these are the same types of cell deaths that have been confirmed in electroporation studies [15].

The presence of HMGB1 as a DAMP molecule has been confirmed in in vitro and in vivo electroporation studies [24,30,31,32,33,34,35]. Nevertheless, whether the release of HMGB1 depends on pulse duration or pulse type remains unknown. Our result demonstrates that HMGB1 can only be released when nanosecond pulses are used (Figure 2). Interestingly, these are the pulse parameters that are believed to affect the membranes of intracellular organelles, including the nucleus, and trigger intracellular effects in addition to increasing permeability of the cell plasma membrane, whereas longer micro- and millisecond pulses have more profound effects on the cell plasma membrane [13,14].

While HMBG1 was present in all cell lines exposed to 200 ns pulses, it was only present at 20% survival, i.e., only when cells were exposed to the electric field leading to the lowest survival (20%). Nonetheless, in the H9c2 cell line, HMGB1 was observed even when survival was barely affected and permeabilization was more prominent. This may indicate that H9c2 has higher assay sensitivity or some HMGB1 can be released due to transient changes in permeability. Increasing electric field strength to 50% led to higher HMGB1 release, possibly from both permeabilized and damaged/dead cells. It is possible that this occurs in all cell lines but is just not as evident as in H9c2 or that the mechanism of HMBG1 release is cell type dependent.

Calreticulin is a protein normally located in the endoplasmic reticulum. To act as a DAMP molecule, it must be actively transferred to the outer leaflet of the plasma membrane [50]. Since the kinetics of calreticulin externalization remain unknown, it was investigated at 4 and 24 h after pulse application, as in the previous study [31] and as in HMBG1. No calreticulin was identified after 4 h, and this goes for all three cell lines. In addition, no calreticulin was identified after 24 h in B16F1 cells, but it was detected in CHO and H9c2 cells (Figure 3). This may indicate that first, the release of calreticulin may have different dynamics in different cell lines and second, it can be cell type dependent. Interestingly, in this study, in CHO cells, calreticulin was only detected 24 h after pulse application, however, in our first study, where CHO cells were used, it was identified as well at 4 h. The same pulses were applied in both studies (8 × 100 μs with 1 Hz repetition frequency). The only difference between experimental settings of the studies was the electrodes. In this newest study, aluminum cuvettes were used, while in our first study, we used stainless steel plate electrodes. The difference in calreticulin externalization due to different electrode material may indicate that DAMP release is not only related to pulse parameters but also to electrochemical reactions occurring on the surface of the electroporation electrodes [51,52,53].

Calreticulin was detected after 24 h in CHO and H9c2 cells after pulse application. It was never identified after millisecond pulse application. Calreticulin was observed when microsecond, nanosecond and HFIRE pulses were used, however, its presence was consistently observed only when pulse amplitudes causing 20% survival were used. Calreticulin observations when pulse amplitudes causing 50% survival were used vary, as it was detected in CHO cells when 4 ns pulses were used and in H9c2 cells when 200 ns and HFIRE pulses were used. It seems that only short pulses (4 ns, 200 ns and 2 μs in HFIRE) caused calreticulin externalization, as longer—microsecond long—pulses had no effect when pulse amplitudes causing 50% survival were used. No calreticulin externalization was detected when pulse amplitudes causing 90% survival (range of reversible electroporation) were used, which is in agreement with our previous study, where we demonstrated a very weak, almost nonexistent correlation between permeability and calreticulin externalization, whereas the correlation between calreticulin externalization and survival was very strong [31].

Both ECT and GET are performed with pulses in the range of reversible electroporation, i.e., trying to avoid irreversible electroporation. Therefore, it is in the range where most of the cell is permeabilized, yet still alive. In our study, this would be pulse amplitudes causing 90% survival (Figure S1). ECT is used for cancer treatment where immune response is favorable, as it can affect the untreated tumor cells as well. The preference for ECT is microsecond pulses. Considering the obtained result of released DAMP molecules in response to microsecond pulse treatment leading to 90% survival, the results show no release of HMGB1 and calreticulin. Only ATP was released, and this can probably be due to changes in cell membrane permeability. Recently, it was shown that ECT can be achieved also with nanosecond and HFIRE pulses and that maybe these can be more immunogenic. Indeed, 200 ns long pulses did cause an HMBG1 release at 90% survival, however, this was observed only in the H9c2 cell line, not in all three cell lines. Therefore, our results strongly suggest that all pulse durations and pulse types are inefficient in inducing DAMP release (except for ATP) when pulses leading to 90% survival are used.

In *in vivo* and clinical settings, there are two other reasons why ECT can lead to immunogenicity of the treatment. The first is irreversible electroporation in close proximity of the electrodes, which occurs due to non-homogeneous electric field distribution. Their effect of pulse treatment is similar to our pulses leading to 20% survival. Interestingly, our study shows that most immunogenic pulse duration is nanosecond pulse treatment, as it can induce release of HMGB1 as well as calreticulin externalization, while traditionally used microsecond pulses only induce calreticulin externalization. Furthermore, we have observed that HFIRE pulses are triggering the same (only calreticulin externalization) in cell as traditional microsecond pulses, indicating that HFIRE pulses could replace monopolar microsecond pulses from the immunogenic perspective. Our results suggest that immune response in ECT can be controlled via pulse duration. The second reason why ECT can lead to immunogenicity is that the treatment consists of pulse application and chemotherapeutic agents. While pulse application in the range of reversible electroporation (leading to 90% survival) may not be enough for immune response activation, chemotherapeutic agents by themselves may be sufficient for activation of immune response. Furthermore, immune response may be activated due to synergistic, i.e., combined effect of electric pulses and chemotherapeutic agents. This was already investigated by Calvet [24], where authors confirmed that this synergistic effect is important for DAMP release in ECT, while electric pulses and chemotherapeutics alone do not always induce DAMP release (HMGB1 was only released in the presence of chemotherapeutics alone, while calreticulin externalization was present after electric pulses alone).

Immune response in GET can be either wanted or unwanted. Similar pulses as in ECT are used also in GET, i.e., pulses leading to 90% survival. GET is traditionally performed using millisecond pulses. Results obtained at 90% survival after millisecond pulse application showed no HMBG1 release or calreticulin externalization, only some ATP release, which we attribute to changes in membrane permeability. Furthermore, recent studies have shown that microsecond, nanosecond and HFIRE pulses can also achieve GET.

Some HMBG1 release was detected when 200 ns pulses causing 90% survival were applied, however, this was detected in only one out of three cell lines. Overall, it seems that all pulse durations and pulse types are inefficient in inducing DAMP release (except for ATP) when pulses leading to 90% survival are used. Therefore, activation of immune response in GET treatment probably occurs due to IRE in proximity of the electrodes due to inhomogeneous electric field distribution as described before. It seems that different pulse durations and types can have different effects on immune response. We have observed that nanosecond pulse treatment is the most immunogenic pulse, as it can induce the release of all three main DAMP molecules, i.e., ATP, HMGB1 and calreticulin. Medium immunogenic pulses are microsecond and HFIRE pulses, as they induce the release of two DAMP molecules—ATP and calreticulin. The least immunogenic pulse seems to be millisecond pulses, as only ATP release was detected and even that we assume occurs due to increased permeability of the cell membrane. Our results suggest that immune response in GET can be controlled via pulse duration. Therefore, nanosecond pulses should be used when immune response is desired and millisecond pulses when immune response is undesired.

Nevertheless, GET is a combination of electrical pulses and genetic material. DNA is normally located in the nucleus and mitochondria, while DNA in the cytosol is recognized as a danger signal. Normally, low levels of DNA can be present in the cytosol, for example, during replication, but DNases digest misplaced DNA to level below the threshold for danger signaling [54]. However, when high amounts of exogenous DNA appear, the cytoplasm threshold seems to be surpassed and DNA sensors are triggered, leading to production of inflammatory cytokines, thus immune response [55]. This may indicate that electroporation pulses alone in the reversible range of amplitudes are not responsible for activation of immune response in GET, as it can be caused by the DNA used in the treatment or there is a synergistic effect of pulses and DNA. However, for now, this remains to be elucidated.

In GET, gene material introduced has to pass a few obstacles [3]. First, genetic material has to cross the cell membrane, then cytosol and at last the nuclear membrane. Interestingly, nanosecond pulses caused the release of HMGB1, which is a nuclear protein. This could mean that nanosecond pulses did indeed permeabilize as well the nuclear membrane, which could be beneficial for GET. Permeabilization of the nuclear membrane would ease the transport of DNA into the cell nucleus, therefore improving the efficiency of GET and improving transgenic protein level expression, which was, however, previously unsuccessfully tested [56].

IRE is used for tumor ablation or heart tissue ablation for treating arrhythmias, such as atrial fibrillation [12]. While activation of the immune system is highly desired and needed in tumor treatment, it is unwanted in cardiac tissue ablation. The inflammation and edema caused by cardiac tissue damage can lead to early recurrence of treated arrhythmia. Due to inflammation, abnormal uneven fibrosis can be present, which can act as an anatomic structure triggering arrhythmias [27,57]. At first, IRE was performed with monopolar microsecond pulses, but recently this was vastly substituted with HFIRE pulses, as they are believed to be less painful [58]. Nevertheless, IRE can be achieved as well with millisecond and nanosecond pulses [15,22,23], however, how different pulse durations and pulse types influence immune response in IRE was not known. For IRE, pulses leading to the lowest survival would be used. In our study, these were pulses leading to 20% survival. It seems that different pulse durations and types can have different effects on immune response, suggesting that immune response in IRE can be controlled via pulse duration. We have observed that the least immunogenic pulses are millisecond pulses, as neither HMGB1 nor calreticulin were detected. This would suggest that millisecond pulses are the most appropriate pulse duration for cardiac tissue ablation. However, it is known that millisecond pulses can cause major electrochemical reactions, leading to bubble formation, which is of a concern, therefore making millisecond pulses inappropriate for such therapy. Nevertheless, millisecond pulses were used even in clinical trials [59,60]. The medium immunogenic pulses seem to be microsecond and HFIRE pulses, as they can induce only calreticulin externalization. The most immunogenic pulses seem to be nanosecond pulses, as they can induce both HMGB1 release and calreticulin externalization.

## 5. Conclusions

In our study, we investigated whether the traditionally used pulse parameters for different electroporation-based therapies are appropriate from the standpoint of immune system activation. We demonstrated that DAMP release and possibly the immune response can be controlled by pulse parameters, such as pulse durations and pulse types. Results show that DAMP release can be different when different pulse durations are used. While millisecond pulses were shown to be the least immunogenic, the most immunogenic were nanosecond pulses. This could be due to changes in permeability of the nuclear membrane and affecting intracellular structures. Overall, it seems that DAMP release and immune response can be manipulated through pulse duration and pulse type. We need to emphasize that reversible electroporation therapies are usually combined with therapeutical agents (chemotherapeutics and genetic material), which can contribute to the immunogenic response. Our result may contribute to the optimization of pulse parameters (pulse duration and pulse type) for a particular treatment, leading to the improvement of electroporation-based therapies.

## Figures and Tables

**Table 1:** Pulse parameters chosen for DAMP analysis.

Pulse Characteristics	90% Survival B16F1/H9c2/CHO	50% Survival B16F1/H9c2/CHO	20% Survival B16F1/H9c2/CHO
5 ms, 8 pulses, 1 Hz	125 V/100 V/100 V	175 V/175 V/175 V	275 V/225 V/200 V
100 μs, 8 pulses, 1 Hz	225 V/200 V/200 V	375 V/350 V/350 V	475 V/500 V/500 V
2-2-2-2 μs, 32 pulses, 100 burst, 1 Hz (HFIRE)	200 V/200 V/200 V	350 V/350 V/300 V	500 V/500 V/400 V
200 ns, 100 pulses, 10 Hz	1.5 kV/2 kV/2 kV	2.5 kV/2.75 kV/3 kV	3 kV/4 kV/4 kV
4 ns, 500 Hz, 12 kV	1000/1000/1500 pulses	2000/2000/3000 pulses	4000/4000/4000 pulses

## Data Availability

Data are available from the corresponding author on request.

## Supplementary Materials

The following supporting information can be downloaded at: https://www.mdpi.com/article/10.3390/vaccines11061036/s1, Figure S1: Measured voltage and current of different pulse durations and pulse types used for achieving 90% survival of CHO cells. Figure S2: Detection of calreticulin signal. Figure S3: Permeability and survival curves obtained using different pulse durations and pulse types. Figure S4: Temperature measurements during pulse delivery. Figure S5: Kinetics of ATP release.
