# Supplementary material for: Immunogenic Cell Death in Electroporation-Based Therapies Depends on Pulse Waveform Characteristics
Source: Vaccines (Basel). 2023 May 29;11(6):1036. doi: 10.3390/vaccines11061036 (PMC10304444; doi:10.3390/vaccines11061036)
Supplement: Supplementary file 1 [file vaccines-11-01036-s001.zip › vaccines-2340888-supplementary.pdf]

Supplementary Materials

Immunogenic Cell Death in Electroporation-Based Therapies Depends on Pulse Waveform Characteristics

Tamara Polajžer and Damijan Miklavčič *

Faculty of Electrical Engineering, University of Ljubljana, Tržaška 25, 1000 Ljubljana, Slovenia;
tamara.polajzer@fe.uni-lj.si
* Correspondence: damijan.miklavcic@fe.uni-lj.si

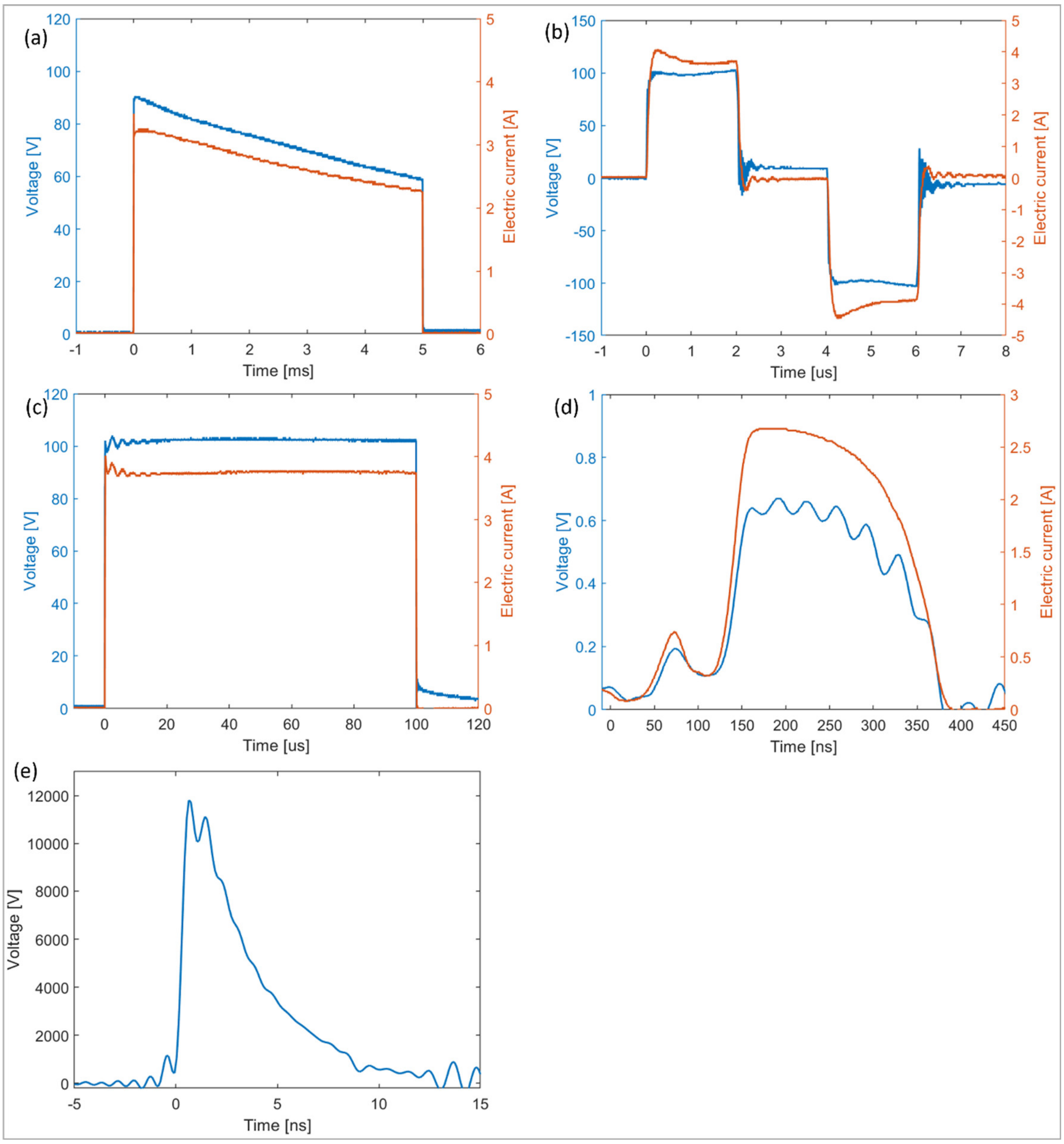

Figure S1. Measured voltage and current of different pulse durations and pulse types used for achieving 90% survival of CHO cells. Voltage is presented in blue, current in red (except for 4 ns pulses, where current was not measured). (**a**) 5 ms, (**b**) HFIRE (2-2-2-2 μs), (**c**) 100 μs, (**d**) 200 ns, (**e**) 4 ns.

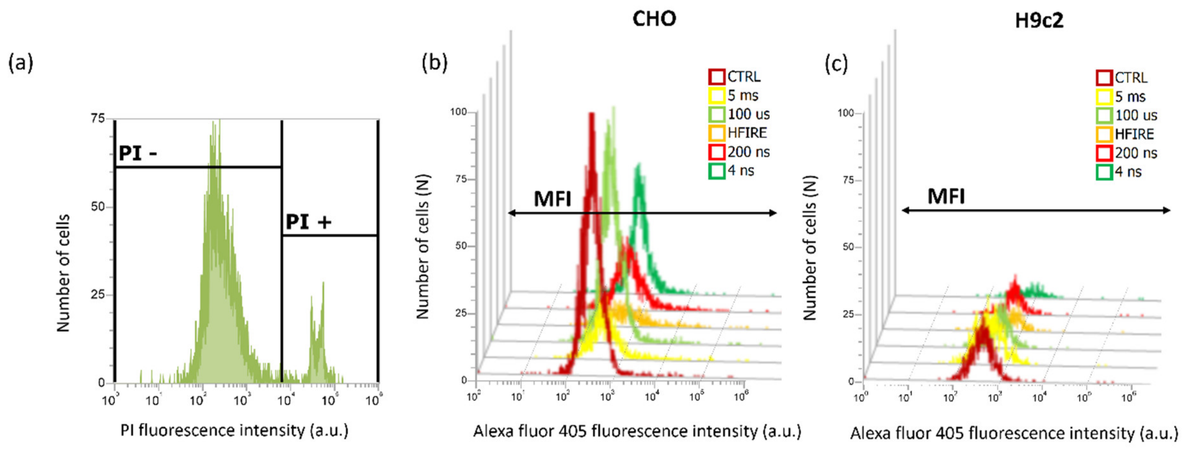

Figure S2. F. Detection of calreticulin signal 24 hours after pulse application. Signal of calreticulin (calreticulin antibody was marked with Alexa fluor 405, this signal was measured only on live (PI-) cells, to detect only externalized calreticulin. Due to low shift in fluorescence, median fluorescence values (MFI) were analyzed. (**a**) Detection of live cells (PI -, negative) for both CHO and H9c2.; (**b**) Alexa fluor 405 (calreticulin) fluorescence intensity in CHO live cells after application of pulses leading to 20% survival; (**c**) Alexa fluor 405 (calreticulin) fluorescence intensity in H9c2 live cells after application of pulses leading to 20% survival.

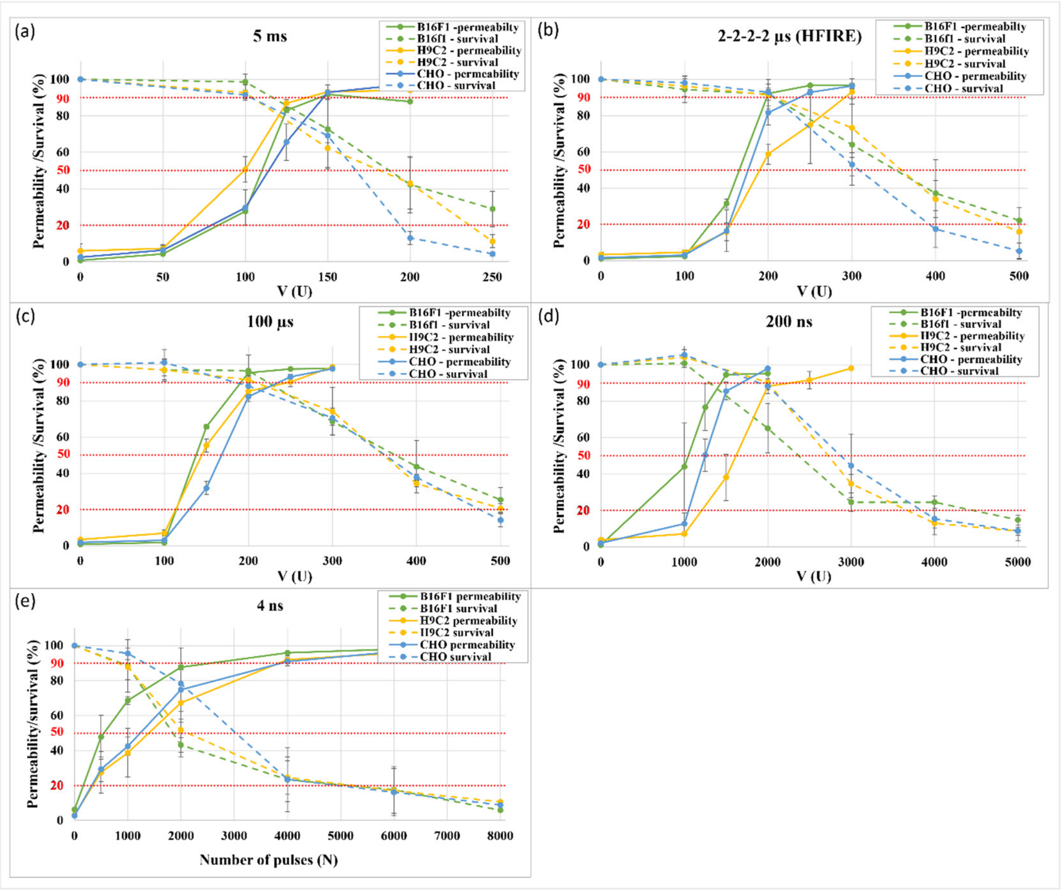

Figure S3. Permeability and survival curves obtained using different pulse durations and pulse types. Experimental points for DAMP analysis were determined based on survival curves, where the curves intersected 90, 50 and 20 % survival (shown with red dotted line). (**a**) 5 ms, (**b**) HFIRE (2-2-2-2 µs), (**c**) 100 µs, (**d**) 200 ns, (**e**) 4 ns.

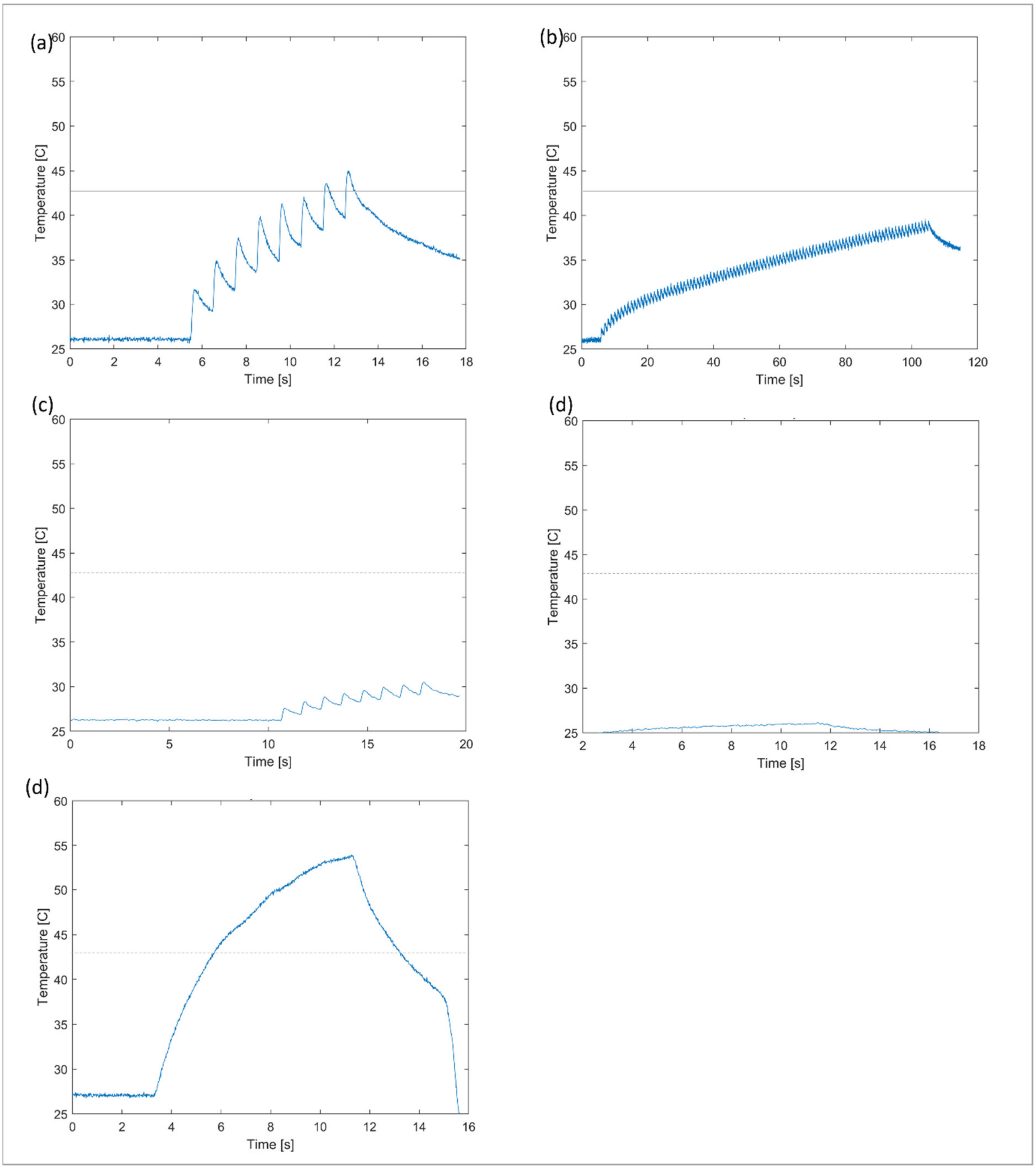

Figure S4. Temperature measurements during pulse delivery. (**a**) 5 ms, (**b**) HFIRE (2-2-2-2 μs), (**c**) 100μs, (**d**) 200 ns, (**e**) 4 ns. Dotted line (. . . . .) represents critical temperature threshold at 43 °C.

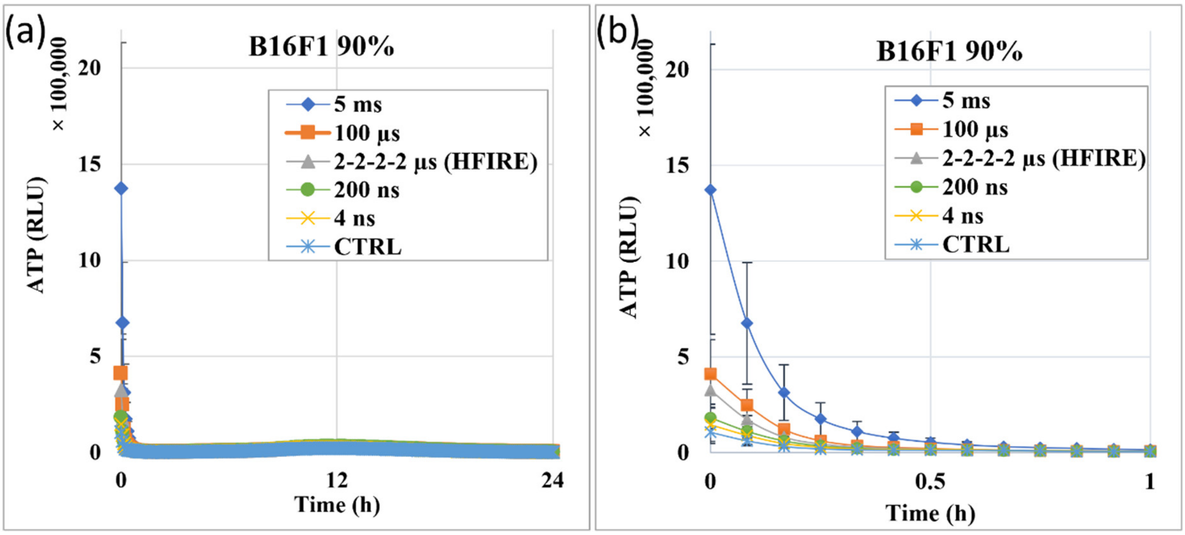

Figure S5. Kinetics of ATP release. Results are shown for cell line B16F1, for cells exposed to pulse amplitudes causing 20% of survival. (**a**) Kinetics of ATP release for 24 hours after pulse application.; where signal was measured every 5 minutes. (**b**) Zoomed kinetic in the first hour after pulse application.
